# Modeling the number of new cases of childhood type 1 diabetes using Poisson regression and machine learning methods; a case study in Saudi Arabia

**DOI:** 10.1371/journal.pone.0321480

**Published:** 2025-04-25

**Authors:** Ahood Alazwari, Laleh Tafakori, Alice Johnstone, Mali Abdollahian

**Affiliations:** 1 School of Science, RMIT University, Melbourne, Victoria, Australia; 2 School of Science, Al-Baha University, Al-Baha, Saudi Arabia; Northeastern University, UNITED STATES OF AMERICA

## Abstract

Diabetes mellitus stands out as one of the most prevalent chronic conditions affecting pediatric populations. The escalating incidence of childhood type 1 diabetes (T1D) globally is a matter of increasing concern. Developing an effective model that leverages Key Performance Indicators (KPIs) to understand the incidence of T1D in children would significantly assist medical practitioners in devising targeted monitoring strategies. This study models the number of monthly new cases of T1D and its associated KPIs among children aged 0 to 14 in Saudi Arabia. The study involved collecting de-identified data (n=377) from diagnoses made between 2010 and 2020, sourced from pediatric diabetes centers in three cities across Saudi Arabia. Poisson regression (PR), and various machine learning (ML) techniques, including random forest (RF), support vector machine (SVM), and K-nearest neighbor (KNN), were employed to model the monthly number of new T1D cases using the local data. The performance of these models was assessed using both numbers of KPIs and metrics such as the coefficient of determination ($R^{2}$), root mean squared error (RMSE), and mean absolute error (MAE). Among various Poisson and ML models, both model considering birth weight over 3.5 kg, maternal age over 25 years at the child’s birth, family history of T1D, and nutrition history, specifically early introduction to cow milk and model taking into account birth weight over 3.5 kg, maternal age over 25 years at the child’s birth, and nutrition history (early introduction to cow milk) emerged as the best-reduced models. They achieved $R^{2}$ of (0.89,0.88), RMSE (0.82, 0.95) and MAE(0.62,0.67). Additionally, models with fewer KPIs, like model that considers maternal age over 25 years and early introduction to cow milk, achieved consistently high $R^{2}$ values ranging from 0.80 to 0.83 across all models. Notably, this model demonstrated smaller values of RMSE (0.92) and MAE (0.67) in the KNN model. Simplified models facilitate the efficient creation and monitoring of KPIs profiles. The findings can assist healthcare providers in collecting and monitoring influential KPIs, enabling the development of targeted strategies to potentially reduce, or reverse, the increasing incidence rate of childhood T1D in Saudi Arabia.

## Introduction

Type 1 diabetes (T1D) is an autoimmune disease that develops as a result of destruction in *β* cells and progression to insulin deficiency [1]. T1D is one of the most common chronic diseases among children and young adults, and its incidence has risen globally in recent decades [2,3]. The rise in incidence has been about 3% annually [4], and the disease currently affects 651,700 children worldwide [5]. The International Diabetes Federation (IDF), in its Atlas’ 10th edition (2022), reported that more than 98,000 children are diagnosed with T1D annually [5]. In addition, it is anticipated that approximately 108,300 children under the age of 15 will be diagnosed with T1D each year, and this number is expected to increase to 128,900 when the age range is extended to 20 years [5]. It is estimated that 3,800 new cases of T1D are diagnosed in children and adolescents in Saudi Arabia each year [5]. Saudi Arabia has a high incidence rate of new cases of T1D in children younger than 15 years old each year (31.4 cases per 100,000 children). Environmental factors are believed to have a role in initiating the autoimmune response, which ultimately leads to the destruction of pancreatic *β* cells destruction, and the development of T1D [6]. The development of T1D has been linked to a wide variety of factors, including infections during childhood, nutrition, factors during pregnancy, and a history of diabetes in relatives [7–9]. Several studies investigated the association between the method of childbirth and the risk of T1D in children, concluding that children born via the caesarian section had a greater risk of developing T1D than children born via normal delivery[10–13]. For gestational age, pre-term (33–36 weeks) [13,14] and early term (37–38 weeks) [13,14], were linked to an increased risk of developing T1D [15]. Maternal characteristics such as advanced maternal age at childbirth [11,16,17] and a mother’s history of diabetes (T1D, T2D, or gestational diabetes) [15,18] have been suggested as risk factors for developing T1D in children. Other maternal health concerns, such as asthma and pre-eclampsia, correlate with an increased risk of T1D in children [15,19]. For child characteristics, a higher birth weight of the child has also been shown to increase T1D risk [13,20]. Children born weighing 3.5 to 4.0 kilograms (kg), or more, had a 6% and 10% diabetes risk increase, respectively [20]. Birth order also affects T1D risk [21,22], firstborn children had the highest risk, which decreased with birth order. Recent reviews of the child’s nutrition indicated that early cow milk exposure might promote T1D [23,24].In the preventative studies [25,26], it was shown that the elimination of cow’s milk proteins in infant formula (in the Finish TRIGR pilot research [25]) or the elimination of bovine insulin in infant formula (in the FINDIA study [26]) reduced the production of islet autoantibodies. Other studies linked the increased rate of T1D in children to their place of residence. In Taiwan, urban living was linked to T1D [27]. Researchers from Finland, Scotland, and Germany found lower incidence rates in urban areas than in rural areas [28–30]. The potential connection between cow milk exposure and T1D, along with successful preventive measures found in studies like the Finish TRIGR pilot and the FINDIA study, calls for a more thorough investigation. Also, the connections between T1D rates, where people live, and economic factors give a broader view of the research. Hence, one of the most important areas of research to focus on is the possibility of delaying, reducing, or preventing complications associated with T1D incidence in children [31–33]. By understanding these factors, we can devise more focused and efficient approaches for managing T1D in children. However, the existing T1D research, such as those conducted in Sweden and Finland [34,35] do not represent the ethnicity and diversity of the Saudi Arabian population. This study aims to fill the gap by examining the number of new cases of TID using data from Saudi Arabia. The proposed work differs from previous research in that it models the the number of new cases of T1D in children by using statistical and machine learning models of its significant risk factors.

### Motivation and the objective of this study

Despite the significant increase in the incidence of T1D in Saudi Arabian children [36–38], current studies conducted in other countries such as Australia, Sweden, and Finland may not adequately reflect the cultural differences and diversity of the Saudi Arabian population. Furthermore, when compared to other developed countries, there is a significant lack of research on T1D specifically in Saudi Arabian children. This highlights a critical gap in our understanding of the disease within the Saudi Arabian context, emphasizing the urgent need for region-specific studies to address the rising the number of new cases and develop tailored prevention and management strategies. Most of the published studies on TID in children in Saudi Arabia are cross-sectional, have small sample sizes, and involve only a single center as well as a single city or region of the country [37]. Two studies only in Saudi Arabia reported the incidence rates of T1D in children [39,40] at higher rates than the IDF estimate. However, these studies were conducted in 2010 and 2011 in a single city or region. In [39], they included children aged from less than 15 years old and only four variables, including gender, age, presentation as diabetic ketoacidosis (DKA), and season of the diagnosis. In [40], they considered children up to 12 years old, and data were analysed according to age, gender and month of presentation. Improvements in Saudi Arabian T1D research are being made with recent studies conducted over three different regions exploring age at onset of T1D in children and identifying the key performance indicators of T1D in children [41,42]. Building upon the KPIs study presented in [42], this research investigates the rising incidence rate of childhood T1D in Saudi Arabia. This study incorporates multiple key factors such as nutrition history, family history of T1D (first and second relative degree), child weight at birth, and maternal age at childbirth. These aspects will contribute to a more comprehensive model for understanding the increasing the number of new cases of T1D in children. To ensure a representative analysis of the country’s vast and diverse population, a multiple-center (cross-sectional) study approach is employed. Utilising Poisson regression and machine learning (ML), this study models the the number of new cases of T1D cases in Saudi Arabian children, leveraging the KPIs identified previously [42]. Through a better understanding of the the number of new cases of T1D and its KPIs, the potential exists to mitigate the disease’s occurrence, thereby contributing to enhancements in the nation’s overall health. Notably, this research broadens the scope of T1D investigation by introducing additional KPIs and incorporating a more diverse population, contributing to the existing body of knowledge in T1D research.

## Materials and method

### Data collection

De-identified data of 377 childhood T1D cases diagnosed between 2010 and 2020 from three different cities (Al-Ahsa, Jeddah, and Riyadh) located in different regions of Saudi Arabia were included in this study. Ethics approval was granted by the RMIT University Human Research Ethics Committee in Australia and the Research Ethics Committee of the Ministry of Health in Saudi Arabia. This was a retrospective study and to collect existing data from medical records, the ethics committee waived the requirement for informed consent. For the additional information collected via a survey of the parents of each child for their residency, income status, and nutritional history, informed consent was obtained, as previously reported. All data were collected and reviewed by trained medical professionals and then were fully anonymised before analysis. The data was accessed on June 25, 2020.

### Dependent variable and independent variables

Each row in our dataset represents a specific year and month, with the dependent variable (Y) being the number of cases of T1D recorded that month. The values for the independent variables correspond to their total sum for the given month. A simulated example of the dataset structure is provided in S1 Table in the Appendix for reference. For this study, data were collected over the period from 2010 to 2020. The significant KPIs identified in the previous study [42] were used as independent variables. The KPIs included in this study are the city, nutrition history, having a family history of T1D (first and second relative degrees), child weight at birth, and maternal age at childbirth.

### Poisson regression and ML models

Most studies on T1D in children use one method to model the cases of this disease, focusing on a few factors. However, employing different and comparable approaches can improve the ability to find the best model for the data, potentially making the number of new cases modeling more accurate. Machine learning can help understand the complex relationships between inputs and outcomes. These methods are flexible, handle many variables without reducing complexity, and prevent overfitting through validation. In this study, we use four different methods — Poisson regression (PR), random forest (RF), support vector machine (SVM), and k-nearest neighbor (KNN) to find the most suitable model for understanding T1D the number of new cases in Saudi Arabian children using its KPIs. Machine learning models have been applied in various aspects of health including diabetes[41–45]. We compare these models with the traditional Poisson regression to better model T1D the number of new cases in children. Moreover, Leave-one-out Cross-Validation (LOOCV) was applied to assess the performance of both Poisson regression and machine learning algorithms. LOOCV is ideal for small datasets, as it maximizes data usage and provides an unbiased performance estimate. It evaluates the model’s ability to generalize by testing each observation individually and helps identify outliers or influential data points. This method is robust against overfitting and ensures a fine-grained assessment of model performance. Furthermore, in the case of Poisson regression, we implemented bootstrapping, a statistical technique involving the resampling of a singular dataset to generate simulated samples and calculate the corresponding standard error [46]. Additionally, to enhance modeling, interactions between variables have been incorporated in Poisson regression and machine learning models, as has been deployed in previous studies [41,43,47–50]. We conducted the analysis using R statistical software (version 4.4.2)[51], using several R packages, including boot, caret, e1071, AER, and randomForest. All code for data analysis is available at https://github.com/Alazwari/R-code.git.

#### Poisson regression model (PR).

Interest in modeling count data has increased significantly over the past two decades [52]. The Poisson distribution is still the most widely used distribution for modeling count data in many research areas [53]. Poisson regression will be used to quantify the relationships of changes in the cases of T1D in children due to changes in significant KPIs. Poisson regression has been used in diabetes research to model the incidence of diabetes[3,39,40,54–56]. Poisson regression method involves expressing the natural logarithm of the event or outcome over a given period of time as a linear function of independent variables.

Poisson log-linear model with the explanatory variable *Y* and independent variables *x*’s is represented by the function


$$Log(Y)=\beta_{0}+\beta_{1}x_{1}+\beta_{2}x_{2}+\ldots +\beta_{K}x_{K}$$

Where β represents the coefficient of the factors and *x* represents the independent variables (IVs).

#### Random forest (RF).

Random forest (RF) is an effective machine-learning approach to improving prediction accuracy and model interpretation[57]. RF methods deal with both supervised classification and regression tasks. RF is a “data-driven statistical method [58].” It is an ensemble learning approach developed to increase classification accuracy and regression tree prediction by combining many decision trees [58]. RF commences using many bootstrap samples randomly drawn with replacements from the original training dataset [58]. Due to its built-in feature selection method, RF can handle a large number of input variables without the need to reduce dimensionality [59]. Also, Out-of-bag validation can be used in RF to prevent overfitting [59].

#### Support vector machine (SVM).

Support vector machine is a machine learning method introduced by Vapnik [60]. In [60], they presented the theory of the optimum hyperplane as a linear classifier and presented nonlinear classifiers through the use of kernel functions. Support vector machine models are classified into support vector machine classifier models and support vector regression models. A support vector machine model is used for resolving data classification problems, and the support vector regression model is used to solve prediction problems. Regression is used to find a hyperplane that fits the given data [60]. In this study, we will use the common kernel, which is the radial basis function (RBF). For this kernel, cross-validation is used to select the value of the parameters that optimise the SVM model. RBF kernel requires the optimisation of two parameters; cost and gamma. The parameter cost controls the over-fitting of the model, and gamma controls the degree of non-linearity of the model [42].

#### K-Nearest Neighbor (KNN).

K-nearest neighbour (KNN) is a well-known machine learning method that has recently been used for the classification and parametric estimation analysis of difficult-to-evaluate unknown probabilities [61,62]. KNN regression predicts the target value by averaging the values of its K nearest neighbours. In this method, the "K" represents the number of neighbouring data points considered in the prediction. KNN regression is a non-parametric approach that doesn’t make any assumptions about the underlying data distribution [61,62]. The idea behind KNN is to sort individual data so that the majority of it comes from the closest neighbour [61,62]. The KNN algorithm is used for both classification and regression to make predictions. In classification, it groups data into categories, while in regression, it uses existing data to predict future values[61,62].

Therefore, in this study, we will use Poisson regression and Machine Learning, Random Forest, Support vector machine and K-Nearest Neighbor to model the monthly number of new cases of T1D in children in Saudi Arabia (2010–2020) in terms of its significant KPIs confirmed by a previous study [42].

### Performance evaluation measures

The common evaluation measures suitable for comparison of regression models are root mean squared error (RMSE), mean absolute error (MAE) and coefficient of determination ($R^{2}$). The model, selected on the performance of different evaluation metrics, has the least RMSE or MAE value and the highest $R^{2}$. The formulas for these metrics are:

$$RMSE=\sqrt{\overset{n}{\underset{i=1}{\sum }}\frac{{\left(x_{i}-\hat{x_{i}}\right)}^{2}}{n}}$$

$$MAE=\frac{1}{n}\overset{n}{\underset{i=1}{\sum }}|x_{i}-\hat{x_{i}}|$$

$$R^{2}=1-\frac{\overset{n}{\underset{i=1}{\sum }}{\left(x_{i}-\hat{x_{i}}\right)}^{2}}{\overset{n}{\underset{i=1}{\sum }}{\left(x_{i}-\bar{x}\right)}^{2}}$$

where $x_{i}$, $\hat{x_{i}}$, and $\bar{x}$ are the observed, predicted and mean values, respectively.

The model with the highest $R^{2}$ and the smallest RMSE and MAE is classified as the best performing model.

## Results

A total of 377 cases from different cities were included in this study, and the characteristics of this study population (children with T1D) are described in Table 1.

**Table 1:** Characteristics of the study participants with their corresponding frequency count and percentage.

Variables	n (%)	Variables	n (%)
Demographic		Environmental	
City: Al-Ahsa	109 (28.91)	Nutritional history: Breastfeeding	64 (16.97)
Jeddah	159 (42.18)	Introduction to cow’s milk	82 (21.75)
Riyadh	109 (28.91)	Both	231 (61.27)
Gender: Male	157 (41.64)	**Obstetric history**	
Female	220 (58.36)	Birth delivery mode: Normal delivery	261 (69.23)
Residency: Rural	119 (31.56)	Cesarean section (CS)	116 (30.77)
Urban	258 (68.43)	Weight at birth: <2.5 kg	115 (30.50)
**Socioeconomic status**: High income	8 (212)	(2.5 - 3.0)kg	168 (44.56)
Upper-middle income	19 (5.03)	(3.0 - 3.5)kg	51 (13.53)
Middle income	305 (80.90)	(3.5 - 4.0)kg	26 (6.89)
Lower-middle income	34 (9.01)	>4.0 kg	17 (4.51)
Low income	11 (2.91)	Birth order: 1st	120 (31.83)
**Genetic**		2nd	70 (18.56)
First-degree of T1D: Yes	85 (22.54)	3rd	74 (19.62)
No	292 (77.45)	Others (4th and more)	113 (29.97)
If Yes, specify: Father	13 (3.44)	**Maternal characteristics**	
Mother	18 (4.77)	Maternal Age at child’s birth: (<25)	80 (21.22)
Sibling	54 (14.3)	Maternal age (25-35)	231 (61.27)
Second-degree of T1D: Yes	95 (25.19)	Maternal age (>35)	66 (17.50)
No	282 (74.80)		

### Diabetes incidence trends

**Fig 1:**
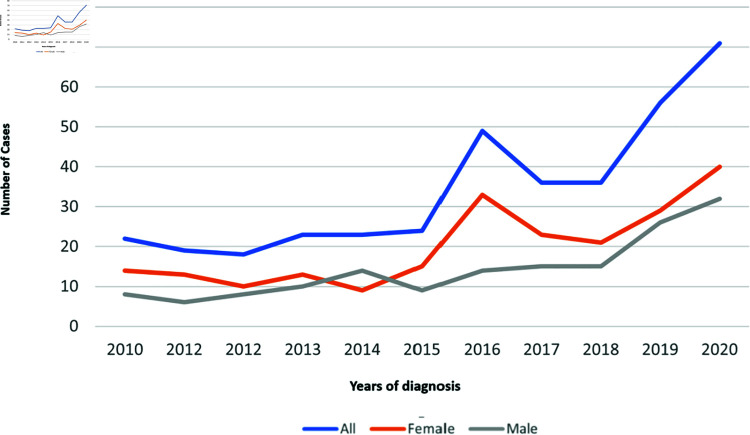
Trend of new cases of childhood T1D by gender during the period (2010–2020).

There was an increase in the number of T1D cases among children aged 0–14 years in Saudi Arabia between 2010 and 2020 (Fig 1). Also, as shown in this figure, the number of T1D cases increased more in females than in males.

### Model development

In this study, we used LOOCV to estimate the performance of Poisson regression and machine learning algorithms. In addition, for Poisson regression, we have used bootstrapping, a statistical procedure that re-samples a single dataset to create simulated samples [46]. To simplify, we have combined some of the levels of variables collected, such as a family history of T1D (combining first and second-degree relatives), child weight (3.5—4.0) kg and (>4) kg, and mother age (25—35 years) and >35 years to be mother age>25 years.

### Poisson regression models (PR)

Poisson regression models were fitted for the target variable, a monthly number of T1D cases, versus independent variables, as shown in Table 2. Model 1 was based on the monthly number of T1D cases and included all significant variables identified in the previous study [42]. These variables are family history of T1D (first and second-degree relatives), nutritional history (early introduction to cow’s milk), nutritional history (mixed), child weight (3.5—4.0) kg and (>4) kg, and mother age (25—35 years) and >35 years to be mother age >25 years, Jeddah city, and rural residency). It is important to note that there are not multiple cases from the same family in our dataset. Then, we aimed to find a simple model (the most parsimonious) to reduce the full model’s complexity and simplify interpretation and monitoring. For further improvement, we considered the interactions between the variables to find the best models.

The results of all fitted Poisson regression models using LOOCV and bootstrapping with 1500 iterations are shown in Table 2. Results indicated that Model 2 performed well when we considered the interactions between variables, and it achieved a high value of $R^{2}$ (0.86) and small values of (RMSE = 0.82 and MAE = 0.62) and $R^{2}$ of (0.89) in the bootstrapping method. Followed by Models 7 and 10 with $R^{2}$ of (0.81 and 0.80) and RMSE of (0.95 and 1.01) and MAE of (0.67 and 0.68) respectively in (LOOCV) and $R^{2}$ of (0.88 and 0.87) in bootstrapping results. In addition, the results showed that models with few variables (Models 8, 9 and 10) performed well in comparison to other models achieving a high $R^{2}$ of (0.86, 0.86, and 0.87) in the bootstrapping method.

**Table 2:** Poisson Regression (PR).

Models	Leave-One-Out Cross-Validation (LOOCV)			Bootstrapping 1500 iterations	
	$R^{2}$	RMSE	MAE	$R^{2}$	std. error
Model 1: cases ∼ child weight>3.5 kg+ Mother age>25+F.H.	0.67	1.63	0.83	0.96	0.006
(2nd degree) + Rural+ F.H.(first degree) +Nutrition (introduction to cow’s milk)					
+ Nutrition (mixed) + City(Jeddah)					
Model 2: cases ∼ child weight>3.5 kg+ Mother age>25+F.H.+	0.64	1.54	0.88	0.89	0.024
Nutrition (introduction to cow’s milk)					
Model 2: cases ∼ child weight>3.5 kg*Mother age>25*F.H.*	0.86	0.82	0.62	0.89	0.022
Nutrition (introduction to cow’s milk)					
Model 3: cases ∼ child weight>3.5 kg+Mother age>25+F.H.(first degree)	0.70	1.27	0.82	0.86	0.28
Model 3: cases ∼ child weight>3.5 kg*Mother age>25*F.H.(first degree)	0.75	1.13	0.76	0.86	0.28
Model 4: cases ∼ child weight>3.5 kg+Mother age>25+F.H.(2nd degree)	0.66	1.45	0.87	0.87	0.027
Model 4: cases ∼ child weight>3.5 kg*Mother age>25*F.H.(2nd degree)	0.70	1.26	0.83	0.87	0.024
Model 5: cases ∼ child weight>3.5 kg+Mother age>25+F.H.	0.63	1.60	0.88	0.87	0.028
(1st and 2nd degree)					
Model 5: cases ∼ child weight>3.5 kg*Mother age>25*F.H.	0.72	1.17	0.75	0.88	0.026
(1st and 2nd degree)					
Model 6: cases ∼ child weight>3.5 kg+Mother age>25+Rural	0.70	1.29	0.84	0.86	0.029
Model 6: cases ∼ child weight>3.5 kg*Mother age>25*Rural	0.72	1.25	0.80	0.87	0.027
Model 7: cases ∼ child weight>3.5 kg+Mother age>25+Nutrition history	0.70	1.30	0.85	0.87	0.025
(Introduction to Cow milk)					
Model 7: cases ∼ child weight>3.5 kg*Mother age>25*Nutrition history	0.81	0.95	0.67	0.88	0.023
(Introduction to cow milk)					
Model 8: cases ∼ child weight>3.5 kg+Mother age>25	0.70	1.29	0.84	0.86	0.028
Model 8: cases ∼ child weight>3.5 kg*Mother age>25	0.68	1.37	0.85	0.86	0.029
Model 9: cases ∼ child weight>3.5 kg+Nutrition history(Introduction to cow’s milk)	0.68	1.35	0.87	0.86	0.025
Model 9: cases ∼ child weight>3.5 kg*Nutrition history(Introduction to cow’s milk)	0.69	1.33	0.85	0.86	0.026
Model 10: cases ∼ Mother age>25+Nutrition history(Introduction to cow’s milk)	0.69	1.36	0.86	0.87	0.024
Model 10: cases ∼ Mother age>25*Nutrition history(Introduction to cow’s milk)	0.80	1.01	0.68	0.87	0.025

*: Interactions between variables, F.H.: Family History of T1D.

*Standard error represents the standard deviation of R-squared corresponding to 1500 bootstrap iterations.

The regression equations for the reduced Poisson regression models (Model 2, Model 7, and Model 9), respectively, are in the appendix (S1 File).

S1–S6 Figs display the dispersion test for the selected Poisson regression models (Model 2, Model 5, Model 7 Model 8, Model 9, and Model 10). The results of the dispersion test used to assess the fitness of the Poisson regression model. The p-value indicates whether there is significant evidence of over-dispersion or under-dispersion in the model. A higher p-value suggests the model adequately fits the data, whereas a low p-value (less than 0.05) indicates potential issues with dispersion that may require alternative models. Additionally, S7–S10 Figs display the plots of actual values versus predicted values for the best Poisson regression models (Models 2, 5, 7, and 10), both with and without interaction terms. Also, multicollinearity has been assessed for Poisson models and the results of the Variance Inflation Factor (VIF). The results presented in S2 Table of the Appendix, show that VIF values (less than 5) indicate that there are no high correlations between independent variables. Also, the confidence intervals for all estimates for regression models 2, 7 and 9 have been provided in S3 Table.

### Machine learning models

The RF, SVM, and KNN were selected as ML methods explored in this study, and their results are presented in Tables 3, 4, and 5. For the RF models, we initially constructed a full model, which demonstrated a high performance with an $R^{2}$ value of 0.92, along with small values of RMSE (0.70) and MAE (0.74). Following this, we investigated reduced models, observing minor changes in performance metrics when considering interactions between variables among the reduced models, Model 2 was the best model, and it achieved the highest value of $R^{2}$ (0.86) and the smallest values of (RMSE = 0.95 and MAE=0.68), followed by Models 7 and 10, which achieved a high $R^{2}$ of (0.82 and 83) and small values of (RMSE =1.06 and 0.96) and (MAE=0.76 and 0.72), respectively. S7–S10 Figs in the appendix present plots of the actual versus predicted values for the best three models both with and without interactions.

**Table 3:** Random Forest (RF).

Models	Leave-One-Out Cross-Validation (LOOCV)		
	$R^{2}$	RMSE	MAE
Model 1: cases ∼ child weight>3.5 kg+ Mother age>25+F.H.	0.92	0.70	0.47
(2nd degree) + Rural+ F.H.(first degree) +Nutrition (introduction to cow’s milk)			
+Nutrition (mixed) + City (Jeddah)			
Model 2: cases ∼ child weight>3.5 kg+ Mother age>25+F.H.+	0.85	0.96	0.69
Nutrition (introduction to cow’s milk)			
Model 2: cases ∼ child weight>3.5 kg*Mother age>25*F.H.*	0.86	0.95	0.68
Nutrition (introduction to cow’s milk)			
Model 3: cases ∼ child weight>3.5 kg+Mother age>25+F.H.(first degree)	0.77	1.14	0.82
Model 3: cases ∼ child weight>3.5 kg*Mother age>25*F.H.(first degree)	0.78	1.13	0.81
Model 4: cases ∼ child weight>3.5 kg+Mother age>25+F.H.(2nd degree)	0.80	1.08	0.83
Model 4: cases ∼ child weight>3.5 kg*Mother age>25*F.H.(2nd degree)	0.80	1.08	0.83
Model 5: cases ∼ child weight>3.5 kg+Mother age>25+F.H.(1st and 2nd degree)	0.84	0.96	0.70
Model 5: cases ∼ child weight>3.5 kg*Mother age>25*F.H.(1st and 2nd degree)	0.83	0.97	0.70
Model 6: cases ∼ child weight>3.5 kg+Mother age>25+Rural	0.78	1.12	0.80
Model 6: cases ∼ child weight>3.5 kg*Mother age>25*Rural	0.78	1.13	0.80
Model 7: cases ∼ child weight>3.5 kg+Mother age>25+Nutrition history	0.82	1.07	0.77
(introduction to cow’s milk)			
Model 7: cases ∼ child weight>3.5 kg*Mother age>25*Nutrition history	0.82	1.06	0.76
(introduction to cow’s milk)			
Model 8: cases ∼ child weight>3.5 kg+Mother age>25	0.79	1.11	0.81
Model 8: cases ∼ child weight>3.5 kg*Mother age>25	0.79	1.10	0.80
Model 9: cases ∼ child weight>3.5 +Nutrition history(introduction to cow’s milk)	0.77	1.12	0.81
Model 9: cases ∼ child weight>3.5*Nutrition history(introduction to cow’s milk)	0.77	1.11	0.81
Model 10: cases ∼ Mother age>25 +Nutrition history(introduction to cow’s milk)	0.83	0.96	0.71
Model 10: cases ∼ Mother age>25 *Nutrition history(introduction to cow’s milk)	0.83	0.96	0.72

*: Interactions between variables; F.H.: Family History of T1D.

For Support Vector Machine (SVM) models, the results also indicated that the full model (Model 1) achieved a high $R^{2}$ of (0.86) and small values of (RMSE =0.76 and MAE = 0.52). Also, Table 4 shows that the reduced models such as Models 2, 5 and Model 10 without interactions between variables were the best models with $R^{2}$ of (0.84, 0.83 and 0.82) and RMSE of (0.89, 0.94 and 0.95) and MAE (0.59, 0.60 and 0.67) respectively. The Radial kernel was used in SVM models, and the parameters of SVM: cost (c) and gamma for these models were (c=1) for both models and gamma = (0.01, 0.3 and 0.5), respectively. By considering the interactions between variables, the best model was Model 10 with a high $R^{2}$ of (0.83) and values of (RMSE=0.93 and MAE=0.66) with (c=1) and gamma of (0.3).

**Table 4:** Support Vector Machine (SVM).

Models	Leave-One-Out Cross-Validation (LOOCV)		
	$R^{2}$	RMSE	MAE
Model 1: cases ∼ child weight>3.5 kg+ Mother age>25+F.H.	0.86	0.76	0.52
(2nd degree) + Rural+ F.H.(first degree) +Nutrition (introduction to cow’s milk)			
+Nutrition (mixed) + City (Jeddah)			
Model 2: cases ∼ child weight>3.5 kg+ Mother age>25+F.H.+	0.84	0.89	0.59
Nutrition (introduction to cow’s milk)			
Model 2: cases ∼ child weight>3.5 kg*Mother age>25*F.H.*	0.82	0.95	0.61
Nutrition (introduction to cow’s milk)			
Model 3: cases ∼ child weight>3.5 kg+Mother age>25+F.H.(first degree)	0.79	1.02	0.68
Model 3: cases ∼ child weight>3.5 kg*Mother age>25*F.H.(first degree)	0.79	1.01	0.66
Model 4: cases ∼ child weight>3.5 kg+Mother age>25+F.H.(2nd degree)	0.76	1.10	0.77
Model 4: cases ∼ child weight>3.5 kg*Mother age>25*F.H.(2nd degree)	0.76	1.09	0.73
Model 5: cases ∼ child weight>3.5 kg+Mother age>25+F.H.(1st and 2nd degree)	0.83	0.94	0.60
Model 5: cases ∼ child weight>3.5 kg*Mother age>25*F.H.(1st and 2nd degree)	0.81	0.97	0.61
Model 6: cases ∼ child weight>3.5 kg+Mother age>25+Rural	0.80	1.01	0.69
Model 6: cases ∼ child weight>3.5 kg*Mother age>25*Rural	0.82	0.97	0.64
Model 7: cases ∼ child weight>3.5 kg+Mother age>25+Nutrition history	0.80	0.97	0.70
(introduction to cow’s milk)			
Model 7: cases ∼ child weight>3.5 kg*Mother age>25*Nutrition history	0.81	0.96	0.71
(introduction to cow’s milk)			
Model 8: cases ∼ child weight>3.5 kg+Mother age>25	0.81	0.99	0.69
Model 8: cases ∼ child weight>3.5 kg*Mother age>25	0.81	0.99	0.72
Model 9: cases ∼ child weight>3.5 +Nutrition history(introduction to cow’s milk)	0.80	1.01	0.70
Model 9: cases ∼ child weight>3.5*Nutrition history(introduction to cow’s milk)	0.80	1.01	0.72
Model 10: cases ∼ Mother age>25 +Nutrition history(introduction to cow’s milk)	0.82	0.95	0.67
Model 10: cases ∼ Mother age>25 *Nutrition history(introduction to cow’s milk)	0.83	0.93	0.66

*: Interactions between variables; F.H.: Family History of T1D.

For the K-Nearest Neighbors (KNN), the number of neighbors (k) is the key parameter. Multiple k-values were used to determine the optimum model. The KNN model with k = 5 revealed the optimal reduced models were Models 2, 5, and 10 with lower RMSE values of (0.86, 0.87, and 0.90), MAE of (0.59, 0.61, and 0.66) and high $R^{2}$ values of (0.86, 0.84, and 0.83). For these selected KNN models, better performance was observed in models without considering interactions between variables. Additionally, the full model with a $R^{2}$ value of (0.93) and smallest values of (RMSE=0.77 and MAE=0.54), demonstrated a high performance.

**Table 5:** K-Nearest Neighbors (KNN).

Models	Leave-One-Out Cross-Validation (LOOCV)		
	$R^{2}$	RMSE	MAE
Model 1: cases ∼ child weight>3.5 kg+ Mother age>25+F.H.	0.93	0.77	0.54
(2nd degree) + Rural+ F.H.(first degree) +Nutrition (introduction to cow’s milk)			
+Nutrition (mixed) + City (Jeddah)			
Model 2: cases ∼ child weight>3.5 kg+ Mother age>25+F.H.+	0.86	0.86	0.59
Nutrition (introduction to cow’s milk)			
Model 2: cases ∼ child weight>3.5 kg*Mother age>25*F.H.*	0.78	1.08	0.78
Nutrition (introduction to cow’s milk)			
Model 3: cases ∼ child weight>3.5 kg+Mother age>25+F.H.(first degree)	0.81	0.95	0.70
Model 3: cases ∼ child weight>3.5 kg*Mother age>25*F.H.(first degree)	0.72	1.16	0.80
Model 4: cases ∼ child weight>3.5 kg+Mother age>25+F.H.(2nd degree)	0.82	0.95	0.70
Model 4: cases ∼ child weight>3.5 kg*Mother age>25*F.H.(2nd degree)	0.72	1.17	0.86
Model 5: cases ∼ child weight>3.5 kg+Mother age>25+F.H.(1st and 2nd degree)	0.84	0.87	0.61
Model 5: cases ∼ child weight>3.5 kg*Mother age>25*F.H.(1st and 2nd degree)	0.80	1.01	0.73
Model 6: cases ∼ child weight>3.5 kg+Mother age>25+Rural	0.82	0.93	0.66
Model 6: cases ∼ child weight>3.5 kg*Mother age>25*Rural	0.71	1.21	0.81
Model 7: cases ∼ child weight>3.5 kg+Mother age>25+Nutrition history	0.83	0.92	0.67
(introduction to cow’s milk)			
Model 7: cases ∼ child weight>3.5 kg*Mother age>25*Nutrition history	0.79	1.03	0.76
(introduction to cow’s milk)			
Model 8: cases ∼ child weight>3.5 kg+Mother age>25	0.82	0.92	0.70
Model 8: cases ∼ child weight>3.5 kg*Mother age>25	0.82	0.93	0.72
Model 9: cases ∼ child weight>3.5 +Nutrition history(introduction to cow’s milk)	0.80	0.93	0.72
Model 9: cases ∼ child weight>3.5*Nutrition history(introduction to cow’s milk)	0.80	0.94	0.74
Model 10: cases ∼ Mother age>25 +Nutrition history(introduction to cow’s milk)	0.83	0.90	0.66
Model 10: cases ∼ Mother age>25 *Nutrition history(introduction to cow’s milk)	0.83	0.92	0.67

*: Interactions between variables; F.H.: Family History of T1D.

## Discussion

This study has several strengths. To the best of our knowledge, it was the largest study to model the number of new cases of T1D in children in Saudi Arabia incorporating a wide range of key performance indicators (KPIs). We have used local data with different statistical and ML approaches to find the best model for the number of new cases of T1D in children in Saudi Arabia using its significant KPIs. De-identified data from 377 children with T1D collected from three cities have been used in this study with different statistical and ML approaches to model the number of new cases of T1D in children in Saudi Arabia. Having access to data including environmental and family history factors of T1D, we have compared the performance of Poisson regression and modern ML approaches to model the number of new cases of T1D. In addition, we used LOOCV methods for Poisson regression and ML approaches to better estimate the performance and the efficacy of the models were assessed using multiple criteria ($R^{2}$, RMSE, and MAE). The model with the highest $R^{2}$ and the smallest RMSE and MAE is classified as the best performing model The results of this study across three cities in Saudi Arabia indicated that the number of new cases of T1D in childhood increased over time from 2013 to 2020 (Fig 1). These results are in alignment with earlier reports from Saudi Arabia, which have also indicated an upward trend in the incidence of T1D among children [39,40]. Similar trends in T1D incidence have also been documented in Sweden [3], Poland [55], and Australia [56]. The analysis showed that the performance of Poisson regression has improved when interactions between variables and bootstrapping are used. Prior research has also utilised Poisson regression model to explore the incidence of T1D. For instance, in Germany, the model was employed to estimate the national T1D incidence and its trends [54]. In Sweden, researchers considered interactions between variables such as year, age, and gender to model T1D incidence effectively [3]. An Australian study used Poisson regression to analyze T1D incidence cases in children, incorporating factors like calendar year, sex, and age group at diagnosis [55]. Conversely, two Saudi Arabian studies conducted in 2010 and 2011 reported T1D incidence rates in children, each with specific criteria for inclusion such as age and limited variables. The authors suggested that incorporating interactions between variables and using Poisson regression has consistently proven beneficial, improving the overall performance of the models. Also, it was used to model the impact of birth weight on the incidence of Type 2 diabetes in youth [63]. As a result of this study, both low and high birth weights were associated with increased risk of Type 2 diabetes in youth (age 10–19 years), while only low birth weight was associated with increased risk in youth (age 20–39 years) [63]. Moreover, ML methods performed well in this study; thus, this technique can be used when modeling count data in agreement with previous studies [64,65]. The result of our study showed that the full model, in Poisson regression based on bootstrapping and in ML, outperformed other models. Models 2 and 7 were the best in Poisson regression of the reduced models when considering interactions between variables. For ML models, again Models 2, 5, and 7 using RF and SVM achieved high $R^{2}$ with only small changes between including or excluding interaction terms. However, in KNN, these reduced models performed better without considering interactions between variables. Additionally, the models with fewer variables (Models 8, 9 and 10) performed relatively well compared to other models in all methods. The variables included in these reduced models were related to the family history of T1D, and maternal or child characteristics. In Model 5, the selected variables encompassed a familial history of T1D (first and second degree), maternal age over 25 years at the child’s birth, and birth weight over 3.5 kg. Model 7 featured nutrition history (initiation of cow milk), maternal age over 25 years at the child’s birth, and birth weight over 3.5 kg. Conversely, Model 8 included maternal age over 25 years at the child’s birth and birth weight over 3.5 kg. For Model 9 birth weight over 3.5 kg and nutrition history (early initiation of cow milk) were considered. For Model 10, maternal age over 25 years at the child’s birth along with nutrition history (early initiation of cow milk) were taken into account. The ML models we developed offer a practical tool for estimating the risk of developing childhood T1D. Healthcare providers could integrate these models into screening tools to identify at-risk populations for closer monitoring or preventive interventions. We also recommend including additional factors such as the mother’s weight so the recommended models can be integrated into electronic health records for automatic risk identification. The findings of this study are in line with earlier studies that demonstrated a relationship between a positive family history of T1D (particularly born to mothers who have T1D) [9,15,18] or early exposure to cow’s milk [23,24] and an increased risk of developing T1D. While it is stated in the literature that the family history of T1D is positively associated with the risk of T1D, to enhance model performance [41],[34],[47-50], we have included interactions between variables. Our results based on interaction reveal a more nuanced relationship. For example, the combination of mother’s age over 25 and cow’s milk exposure reduces the direct effect of family history in Model 2. For early exposure to cow’s milk, other studies also found early cow’s milk exposure and short breastfeeding (2-4 months) may raise susceptibility [24] and T1D was strongly linked to the absence of breastfeeding [66]. Further, most selected models in this study contained maternal age as a KPI, in agreement with previous studies that have linked maternal age over 25 years to T1D risk [67,68]. Comparing maternal ages greater than 35 to those less than 25, the risk of childhood T1D increased significantly in the older maternal age group [16]. In [11], they found that the risk of T1D in children increased by 5% for every five-year increase in maternal age. There seems to be a link between maternal age and autoimmune diseases in children [17]. An indicator of accumulated multiple exposures or pregnancy complications may be maternal age. In addition, a higher birth weight of the child has also been shown to increase T1D risk [13,20]. Children born weighing 3.5 to 4.0 kilograms (kg), or more, had a 6% and 10% diabetes risk increase respectively [20]. However, other maternal characteristics such as gestational diabetes, maternal history of asthma, and pre-eclampsia were not included in this study as they weren’t identified as significant factors of T1D in children in Saudi Arabia in the previous study [42] but were shown as significant factors in other countries [13–15,17]. This may reflect the small number of observations related to these characteristics in [42]. In addition, the mother’s weight at childbirth [69] was not included in the medical records of Saudi Arabian children, which is a limitation of using secondary data in this study. This should be considered as a key factor in future data collection for research, particularly as female obesity has increased in Saudi Arabia over the last decade [70]. The results presented here show the importance of collecting and monitoring significant KPIs to improve public health outcomes. The creation of a unified electronic health record linking all hospitals in the country would increase the efficacy of data collection (sample size, diversity, and monitoring of pregnancy variables, birth characteristics, and child development over time) and enable further refinement of our T1D models. The strength of this study is exploring a range of KPIs of T1D in children to model the number of new cases of T1D using Poisson regression and machine learning methods (RF, SVM and KNN).

## Conclusion

This study marks a significant contribution as the first extensive investigation conducted across various regions to model the number of new cases of childhood T1D in Saudi Arabia, considering both environmental and family history factors. Despite ranking as the 5th highest in T1D incidence rates globally and having the 7th-most T1D children, Saudi Arabia lacks targeted and comprehensive T1D research when compared to developed countries. Prior research on childhood T1D in Saudi Arabia have been limited by factors such as small sample sizes, single-center studies, focus on a specific city or region, or a limited exploration of associated influencing factors. In contrast, this study draws upon data from 377 children with T1D from three cities spanning diverse regions of Saudi Arabia, providing a more representative sample of the country’s population. Additionally, the research incorporates a broad spectrum of previously identified Key Performance Indicators (KPIs). We have utilised statistical and machine learning approaches (RF, SVM, and KNN) to model the number of new cases of childhood T1D using the most influential KPIs. In the healthcare domain, there is a growing interest in the application of Machine Learning methods. The analysis reveals an upward trend in the number of new cases of T1D in children and evidence of a pattern in the number of new cases of childhood T1D by gender. Furthermore, more KPIs identified previously were included to model the number of new cases of T1D in children. Models that include a family history of T1D (first and second degree), maternal age over 25 years at the child’s birth, birth weight over 3.5 kg, nutrition history (early introduction to cow milk) (Model 2), maternal age over 25 years at the child’s birth, and birth weight over 3.5 kg, and nutrition history (early introduction to cow milk) (Model 7) were the best models when comparing the performance of different models. In addition, we have considered the simplified models consisting of maternal age over 25 years at the child’s birth, birth weight over 3.5 kg, and nutritional history (early introduction to cow’s milk) (Models 8, 9, and 10). These models achieved a high $R^{2}$ of (0.86, 0.86, and 0.87) based on the bootstrapping method in Poisson regression and ML models. Models 8, 9, and 10 are simple models with fewer model parameters, which may make it easier for clinicians to interpret compared to an overly complex model. The optimal reduced models (Models 8, 9 and 10) with fewer variables will be used to develop a profile monitoring program for KPIs of T1D in children. By integrating these variables into a multi-faceted approach involving policy development, educational campaigns, and mentoring programs, there is an opportunity to proactively address T1D incidence in children. For instance: developing maternal health policies that advocate for increased access to prenatal care, nutritional support, and family planning services. Infant feeding initiatives should be implemented, emphasising evidence-based practices such as breastfeeding. Additionally, mentoring programs for pregnant women can offer guidance on maintaining a healthy lifestyle, with an emphasis on proper nutrition and prenatal care to optimize birth weight. Supporting healthcare professionals through resources and assistance enables them to monitor and address factors influencing birth weight, contributing to a holistic approach to maternal and child health. This study makes a significant contribution to the T1D literature as well as to Saudi Arabian childhood T1D research by providing the optimal and simplest model to predict the number of new cases of T1D in children. This would enable suitable intervention strategies to reduce the disease burden and potentially slow childhood T1D incidence in Saudi Arabia. In addition, the study demonstrates that having access to a nationwide electronic health record database connected to all of the hospitals in the country would greatly improve health outcomes. This could be utilised to further improve the model’s accuracy regarding the characteristics associated with population diversity, which is considered a limitation of this study. The findings presented in this paper have also contributed towards bridging the research gap in childhood T1D research in non-European nations.

## Supporting information

S1 File Regression equations for the reduced Poisson regression models. (Model 2, Model 7, and Model 9)
(PDF)

S1 Fig Dispersion test for Poisson regression Model 2. Results of the dispersion test for evaluating the fit of the Poisson regression model. The p-value evaluates the the evidence of overdispersion or underdispersion in the model. A p-value above 0.05 suggests adequate model fit, while a p-value below 0.05 indicates potential dispersion issues, warranting consideration of alternative models
(TIF)

S2 Fig Dispersion test for Poisson regression Model 5. Results of the dispersion test for evaluating the fit of the Poisson regression model. The p-value evaluates the the evidence of overdispersion or underdispersion in the model. A p-value above 0.05 suggests adequate model fit, while a p-value below 0.05 indicates potential dispersion issues, warranting consideration of alternative models
(TIF)

S3 Fig Dispersion test for Poisson regression Model 7. Results of the dispersion test for evaluating the fit of the Poisson regression model. The p-value evaluates the the evidence of overdispersion or underdispersion in the model. A p-value above 0.05 suggests adequate model fit, while a p-value below 0.05 indicates potential dispersion issues, warranting consideration of alternative models
(TIF)

S4 Fig Dispersion test for Poisson regression Model 8. Results of the dispersion test for evaluating the fit of the Poisson regression model. The p-value evaluates the the evidence of overdispersion or underdispersion in the model. A p-value above 0.05 suggests adequate model fit, while a p-value below 0.05 indicates potential dispersion issues, warranting consideration of alternative models
(TIF)

S5 Fig Dispersion test for Poisson regression Model 9. Results of the dispersion test for evaluating the fit of the Poisson regression model. The p-value evaluates the the evidence of overdispersion or underdispersion in the model. A p-value above 0.05 suggests adequate model fit, while a p-value below 0.05 indicates potential dispersion issues, warranting consideration of alternative models
(TIF)

S6 Fig Dispersion test for Poisson regression Model 10. Results of the dispersion test for evaluating the fit of the Poisson regression model. The p-value evaluates the the evidence of overdispersion or underdispersion in the model. A p-value above 0.05 suggests adequate model fit, while a p-value below 0.05 indicates potential dispersion issues, warranting consideration of alternative models
(TIF)

S7 Fig Plots of actual versus predicted values for the best Poisson regression models (Models 2, 7, and 10), both with and without interaction. (TIF)

S8 Fig Plots of actual versus predicted values for the best RF models (Models 2, 7, and 10), both with and without interaction. (TIF)

S9 Fig Plots of actual versus predicted values for the best SVM models (Models 2, 5, and 10), both with and without interaction. (TIF)

S10 Fig Plots of actual versus predicted values for the best KNN models (Models 2, 5, and 10), both with and without interaction. (TIF)

S1 Table Simulated example from the dataset showing the first five rows from the year 2015. (DOCX)

S2 Table Variance Inflation Factors for Poisson Regression Model Variables for all models. (DOCX)

S3 Table Confidence Intervals for Models 2, 7 and 9. (DOCX)
