# Supplementary material for: Modeling the number of new cases of childhood type 1 diabetes using Poisson regression and machine learning methods; a case study in Saudi Arabia
Source: PLoS One. 2025 Apr 25;20(4):e0321480. doi: 10.1371/journal.pone.0321480 (PMC12027261; doi:10.1371/journal.pone.0321480)
Supplement: S1 File — (Model 2, Model 7, and Model 9) (PDF) [file pone.0321480.s001.pdf]

Model 2 equation

$$Log(cases) = -0.955 + 0.782(childweight{>}3.5kg) + 0.741(Motherage{>}25) + 0.569 (F.H.) + 0.774(cow'smilk) - 0.319(childweight{>}3.5kg : Motherage{>}25) - 0.320 (childweight{>}3.5kg : F.H.) - 0.186(Motherage{>}25 : F.H.) - 0.133(childweight{>}3.5kg : cow'smilk) - 0.278(Motherage{>}25 : cow'smilk) - 0.201(F.H. : cow'smilk) + 0.123 (childweight{>}3.5kg : Motherage{>}25 : F.H.) + 0.084(childweight{>}3.5kg : Motherage{>}25 : cow'smilk) + 0.177(childweight{>}3.5kg : F.H. : cow'smilk) + 0.084(Motherage{>}25 : F.H. : cow'smilk) - 0.058(childweight{>}3.5kg : Motherage{>}25 : F.H. : cow'smilk)$$

Model 7 equation

$$Log(cases) = -0.495 + 0.624(childweight{>}3.5kg) + 0.550(Motherage{>}25) + 0.678 (cow'smilk) - 0.214(childweight{>}3.5kg : Motherage{>}25) - 0.152(childweight{>}3.5kg : Nutritionhistory) - 0.188(Motherage{>}25 : cow'smilk) + 0.073(childweight{>}3.5kg : Motherage{>}25 : cow'smilk)$$

Model 9 equation

$$Log(cases) = 0.495 + 0.491(childweight{>}3.5kg) + 0.556(cow'smilk) - 0.245(child weight{>}3.5kg : cow'smilk)$$
