# Supplementary figures and images for: Modeling the number of new cases of childhood type 1 diabetes using Poisson regression and machine learning methods; a case study in Saudi Arabia
Source: PLoS One. 2025 Apr 25;20(4):e0321480. doi: 10.1371/journal.pone.0321480 (PMC12027261; doi:10.1371/journal.pone.0321480)

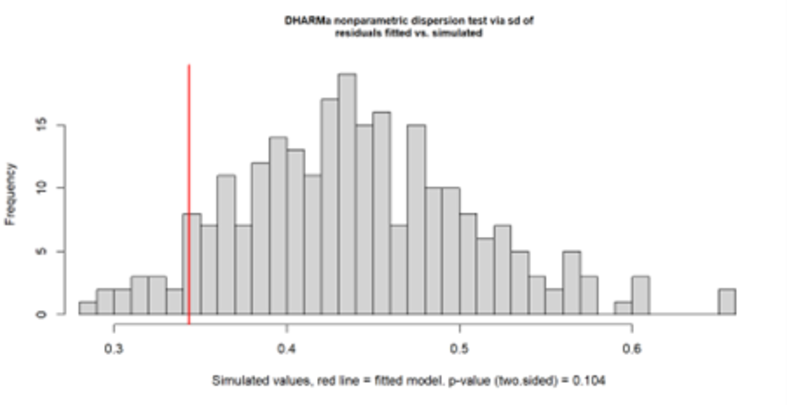

Supplement: S1 Fig — Results of the dispersion test for evaluating the fit of the Poisson regression model. The p-value evaluates the the evidence of overdispersion or underdispersion in the model. A p-value above 0.05 suggests adequate model fit, while a p-value below 0.05 indicates potential dispersion issues, warranting consideration of alternative models (TIF) [file pone.0321480.s002.tif]

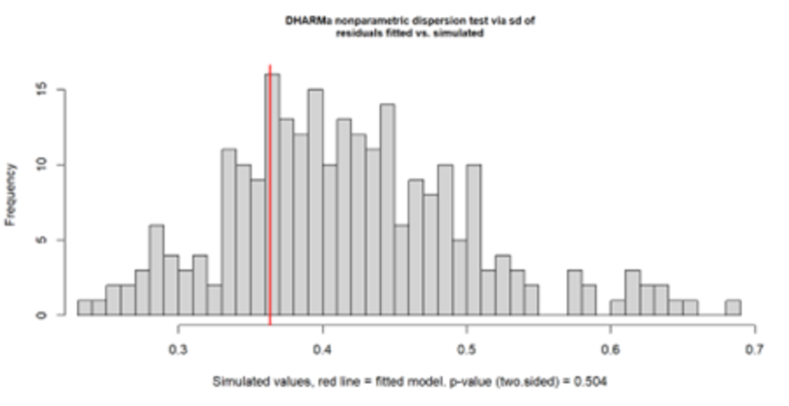

Supplement: S2 Fig — Results of the dispersion test for evaluating the fit of the Poisson regression model. The p-value evaluates the the evidence of overdispersion or underdispersion in the model. A p-value above 0.05 suggests adequate model fit, while a p-value below 0.05 indicates potential dispersion issues, warranting consideration of alternative models (TIF) [file pone.0321480.s003.tif]

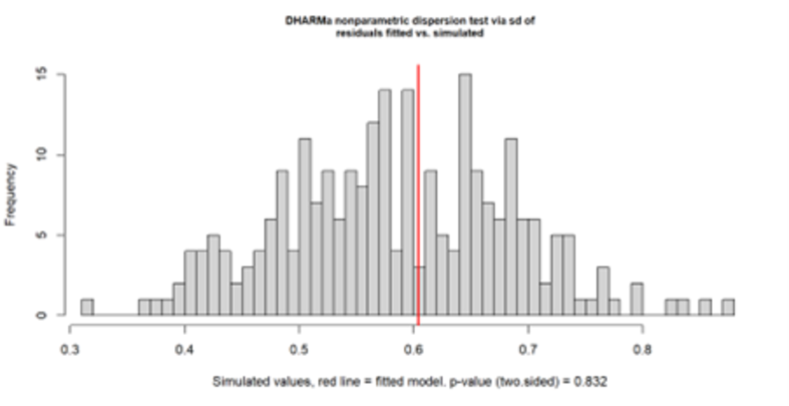

Supplement: S3 Fig — Results of the dispersion test for evaluating the fit of the Poisson regression model. The p-value evaluates the the evidence of overdispersion or underdispersion in the model. A p-value above 0.05 suggests adequate model fit, while a p-value below 0.05 indicates potential dispersion issues, warranting consideration of alternative models (TIF) [file pone.0321480.s004.tif]

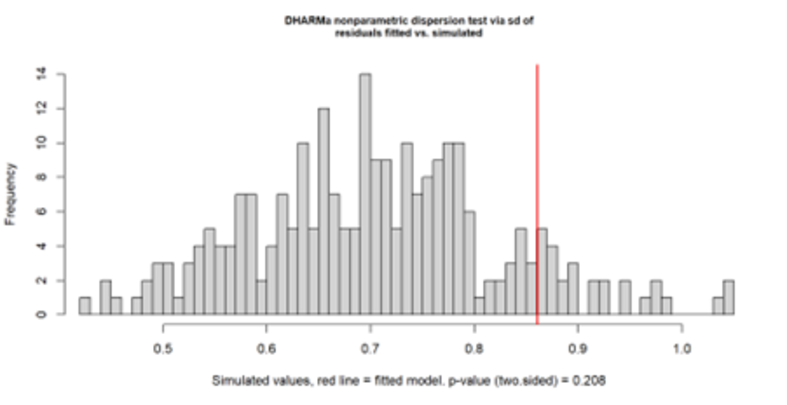

Supplement: S4 Fig — Results of the dispersion test for evaluating the fit of the Poisson regression model. The p-value evaluates the the evidence of overdispersion or underdispersion in the model. A p-value above 0.05 suggests adequate model fit, while a p-value below 0.05 indicates potential dispersion issues, warranting consideration of alternative models (TIF) [file pone.0321480.s005.tif]

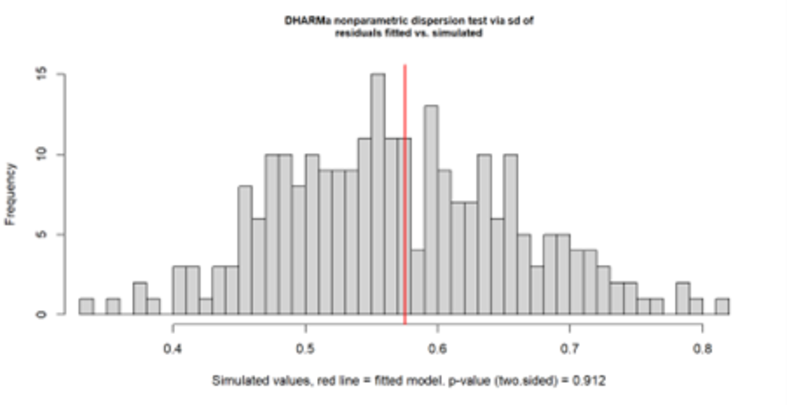

Supplement: S5 Fig — Results of the dispersion test for evaluating the fit of the Poisson regression model. The p-value evaluates the the evidence of overdispersion or underdispersion in the model. A p-value above 0.05 suggests adequate model fit, while a p-value below 0.05 indicates potential dispersion issues, warranting consideration of alternative models (TIF) [file pone.0321480.s006.tif]

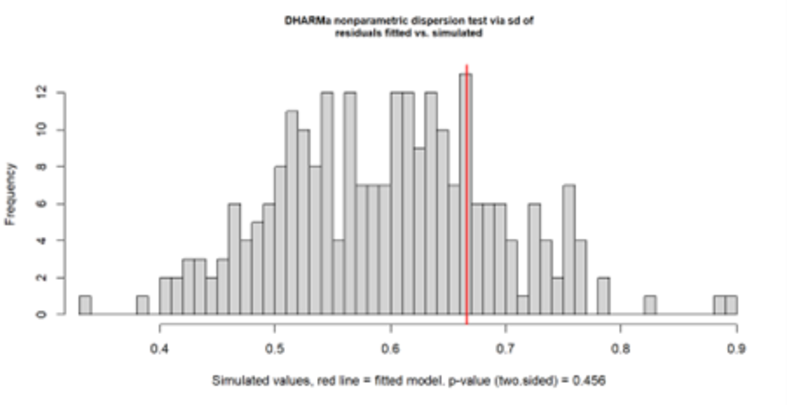

Supplement: S6 Fig — Results of the dispersion test for evaluating the fit of the Poisson regression model. The p-value evaluates the the evidence of overdispersion or underdispersion in the model. A p-value above 0.05 suggests adequate model fit, while a p-value below 0.05 indicates potential dispersion issues, warranting consideration of alternative models (TIF) [file pone.0321480.s007.tif]

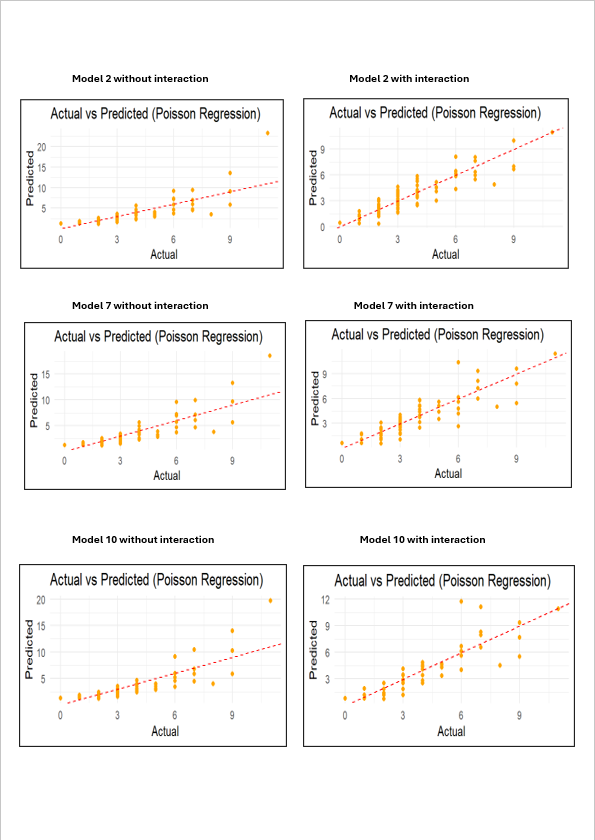

Supplement: S7 Fig — (TIF) [file pone.0321480.s008.tif]

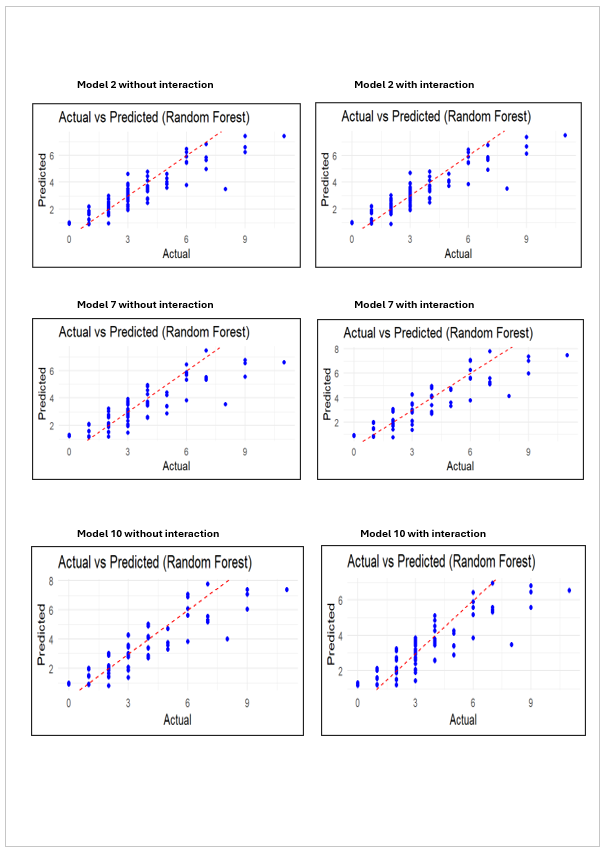

Supplement: S8 Fig — (TIF) [file pone.0321480.s009.tif]

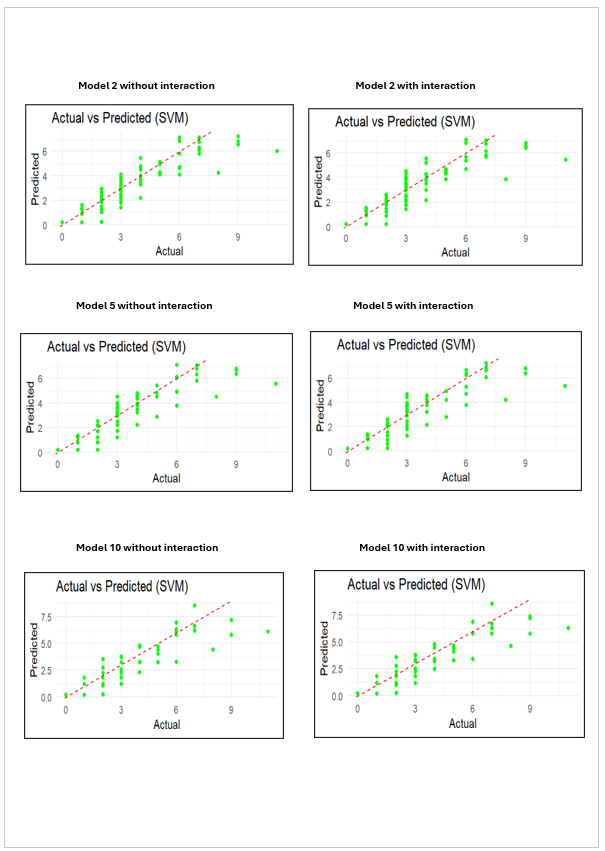

Supplement: S9 Fig — (TIF) [file pone.0321480.s010.tif]

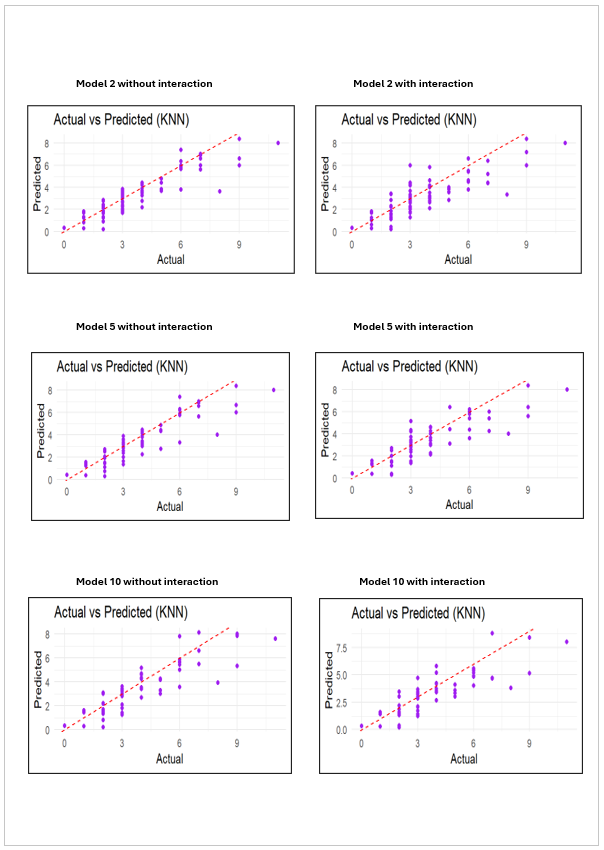

Supplement: S10 Fig — (TIF) [file pone.0321480.s011.tif]
