# Supplementary material for: Modeling the number of new cases of childhood type 1 diabetes using Poisson regression and machine learning methods; a case study in Saudi Arabia
Source: PLoS One. 2025 Apr 25;20(4):e0321480. doi: 10.1371/journal.pone.0321480 (PMC12027261; doi:10.1371/journal.pone.0321480)
Supplement: S1 Table — (DOCX) [file pone.0321480.s012.docx]

S1 Table: Simulated example from the dataset showing the first five rows from the year 2015

| Year | Month | Y (Cases) | City A | City B | City C | Female | Male | Rural | Urban |
| --- | --- | --- | --- | --- | --- | --- | --- | --- | --- |
| 2015 | 1 | 5 | 2 | 1 | 2 | 3 | 2 | 1 | 4 |
| 2015 | 2 | 3 | 1 | 1 | 1 | 1 | 2 | 2 | 1 |
| 2015 | 3 | 4 | 2 | 0 | 2 | 2 | 2 | 1 | 3 |
| 2015 | 4 | 2 | 1 | 0 | 1 | 1 | 1 | 1 | 1 |
| 2015 | 5 | 6 | 3 | 2 | 1 | 4 | 2 | 2 | 4 |
