# Supplementary material for: Modeling the number of new cases of childhood type 1 diabetes using Poisson regression and machine learning methods; a case study in Saudi Arabia
Source: PLoS One. 2025 Apr 25;20(4):e0321480. doi: 10.1371/journal.pone.0321480 (PMC12027261; doi:10.1371/journal.pone.0321480)
Supplement: S2 Table — (DOCX) [file pone.0321480.s013.docx]

**S1 Table. Variance Inflation Factors for Poisson Regression Model Variables for all models**

| Models | Variables | VIF |
| --- | --- | --- |
| Model 1 | Child Weight more 3.5 | 1.109240 |
|  | Maternal Age over 25 | 4.640837 |
|  | Family history of T1D | 1.607819 |
|  | Having.2nd.family.H.T1D | 3.268227 |
|  | Rural | 2.081444 |
|  | Nutrition history “Early introduction to cow milk” | 1.829612 |
|  | Nutrition history “mixed” | 4.789742 |
|  | Jeddah | 4.122990 |
| Model 2 | Child Weight more 3.5 | 1.102109 |
|  | Maternal Age over 25 | 3.339667 |
|  | Family history of T1D | 1.450969 |
|  | Nutrition history “Early introduction to cow milk” | 3.172253 |
| Model 3 | Child Weight more 3.5 | 1.022758 |
|  | Maternal Age over 25 | 1.324753 |
|  | Family history of T1D (first degree) | 1.306204 |
| Model 4 | Child Weight more 3.5 | 1.062548 |
|  | Maternal Age over 25 | 2.298109 |
|  | Family history of T1D (second degree) | 2.266709 |
| Model 5 | Child Weight more 3.5 | 1.090262 |
|  | Maternal Age over 25 | 3.204236 |
|  | Family history of T1D | 3.170467 |
| Model 6 | Child Weight more 3.5 | 1.018229 |
|  | Maternal Age over 25 | 1.684062 |
|  | Rural | 1.661052 |
| Model 7 | Child Weight more 3.5 | 1.038494 |
|  | Maternal Age over 25 | 1.428472 |
|  | Nutrition history “Early introduction to cow milk” | 1.414649 |
| Model 8 | Child Weight more 3.5 | 1.016001 |
|  | Maternal Age over 25 | 1.016001 |
| Model 9 | Child Weight more 3.5 | 1.00018 |
|  | Maternal Age over 25 | 1.00018 |
| Model 10 | Child Weight more 3.5 | 1.405672 |
|  | Maternal Age over 25 | 1.405672 |
