# Supplementary material for: Modeling the number of new cases of childhood type 1 diabetes using Poisson regression and machine learning methods; a case study in Saudi Arabia
Source: PLoS One. 2025 Apr 25;20(4):e0321480. doi: 10.1371/journal.pone.0321480 (PMC12027261; doi:10.1371/journal.pone.0321480)
Supplement: S3 Table — (DOCX) [file pone.0321480.s014.docx]

**S2 Table Confidence Intervals for Models 2, 7 and 9**

| Regression Estimates with Confidence Intervals for Model 2 | | | | | |
| --- | --- | --- | --- | --- | --- |
| term | **estimate** | **conf.low** | **conf.high** | **std.error** | **p.value** |
| (Intercept) | -0.9558116 | -1.5536821 | -0.4343554 | 0.2846777 | 0.0007864 |
| Child weight>3.5 kg | 0.7827318 | -0.4466434 | 1.9030414 | 0.5953916 | 0.1886274 |
| Mother age>25 | 0.7419890 | 0.4833810 | 1.0129577 | 0.1347684 | 0.0000000 |
| FAMILY HISTORY OF T1D . | 0.5695587 | 0.1554587 | 0.9869451 | 0.2115004 | 0.0070824 |
| Nutrition history(introduction to cow’s milk) | 0.7748513 | 0.3250966 | 1.2307476 | 0.2303864 | 0.0007703 |
| Child weight>3.5 kg:Mother age>25 | -0.3195250 | -0.7606992 | 0.1313303 | 0.2266593 | 0.1586238 |
| Child weight>3.5 kg:FAMILY HISTORY OF T1D . | -0.3205662 | -0.9580999 | 0.3207111 | 0.3249964 | 0.3239522 |
| Mother age>25:FAMILY HISTORY OF T1D . | -0.1861159 | -0.3241458 | -0.0537122 | 0.0688565 | 0.0068727 |
| Child weight>3.5 kg:Nutrition history(introduction to cow’s milk) | -0.1335698 | -2.1976829 | 1.7873287 | 1.0092443 | 0.8947103 |
| Mother age>25:Nutrition history(introduction to cow’s milk) | -0.2788420 | -0.4436693 | -0.1211459 | 0.0821524 | 0.0006883 |
| FAMILY HISTORY OF T1D .:Nutrition history(introduction to cow’s milk) | -0.2014730 | -0.4480631 | 0.0400073 | 0.1243137 | 0.1050860 |
| Child weight>3.5 kg:Mother age>25:FAMILY HISTORY OF T1D . | 0.1238916 | -0.0712631 | 0.3161490 | 0.0985849 | 0.2088624 |
| Child weight>3.5 kg:Mother age>25:Nutrition history(introduction to cow’s milk) | 0.0843719 | -0.4561010 | 0.6407602 | 0.2779272 | 0.7614513 |
| Child weight>3.5 kg:FAMILY HISTORY OF T1D .:Nutrition history(introduction to cow’s milk) | 0.1771468 | -0.7015444 | 1.1065542 | 0.4593911 | 0.6997839 |
| Mother age>25:FAMILY HISTORY OF T1D .:Nutrition history(introduction to cow’s milk) | 0.0848039 | 0.0175170 | 0.1549018 | 0.0349817 | 0.0153406 |
| Child weight>3.5 kg:Mother age>25:FAMILY HISTORY OF T1D .:Nutrition history(introduction to cow’s milk) | -0.0587623 | -0.2873998 | 0.1618396 | 0.1140872 | 0.6065079 |
| Regression Estimates with Confidence Intervals for Model 7 |  |  |  |  |  |
| term | **estimate** | **conf.low** | **conf.high** | **std.error** | **p.value** |
| (Intercept) | -0.4954892 | -0.8901068 | -0.1322691 | 0.1931272 | 0.0102994 |
| Child weight>3.5 kg | 0.6247331 | -0.0070782 | 1.2329503 | 0.3156217 | 0.0477740 |
| Mother age>25 | 0.5508641 | 0.4064582 | 0.6995875 | 0.0747252 | 0.0000000 |
| Nutrition history(introduction to cow’s milk) | 0.6781786 | 0.4009514 | 0.9575701 | 0.1418029 | 0.0000017 |
| Child weight>3.5 kg:Mother age>25 | -0.2146458 | -0.4098148 | -0.0190064 | 0.0996018 | 0.0311587 |
| Child weight>3.5 kg:Nutrition history(introduction to cow’s milk) | -0.1522505 | -0.8542885 | 0.5575610 | 0.3595461 | 0.6719655 |
| Mother age>25:Nutrition history(introduction to cow’s milk) | -0.1887323 | -0.2725547 | -0.1073235 | 0.0421312 | 0.0000075 |
| Child weight>3.5 kg:Mother age>25:Nutrition history(introduction to cow’s milk) | 0.0731181 | -0.1087569 | 0.2509146 | 0.0916477 | 0.4249764 |
| Regression Estimates with Confidence Intervals for Model 9 |  |  |  |  |  |
| term | **estimate** | **conf.low** | **conf.high** | **std.error** | **p.value** |
| (Intercept) | 0.4959922 | 0.3097057 | 0.6736459 | 0.0928066 | 0.0000001 |
| Child weight>3.5 kg | 0.4914354 | 0.2742397 | 0.6984426 | 0.1080790 | 0.0000054 |
| Nutrition history(introduction to cow’s milk) | 0.5568826 | 0.4171850 | 0.6946545 | 0.0707415 | 0.0000000 |
| Child weight>3.5 kg:Nutrition history(introduction to cow’s milk) | -0.2458156 | -0.4329866 | -0.0614485 | 0.0946827 | 0.0094260 |
